# Nucleofection of Rodent Neuroblasts to Study Neuroblast Migration *In vitro*

**DOI:** 10.3791/50989

**Published:** 2013-11-12

**Authors:** Katarzyna Falenta, Sangeetha Gajendra, Martina Sonego, Patrick Doherty, Giovanna Lalli

**Affiliations:** ^1^Wolfson Centre for Age-Related Diseases, King's College London; ^2^MRC Centre for Developmental Neurobiology, King's College London

**Keywords:** Neuroscience, Issue 81, Cellular Biology, Cell Migration Assays, Transfection, Neurogenesis, subventricular zone (SVZ), neural stem cells, rostral migratory stream (RMS), neuroblast, 3D migration assay, nucleofection

## Abstract

The subventricular zone (SVZ) located in the lateral wall of the lateral ventricles plays a fundamental role in adult neurogenesis. In this restricted area of the brain, neural stem cells proliferate and constantly generate neuroblasts that migrate tangentially in chains along the rostral migratory stream (RMS) to reach the olfactory bulb (OB). Once in the OB, neuroblasts switch to radial migration and then differentiate into mature neurons able to incorporate into the preexisting neuronal network. Proper neuroblast migration is a fundamental step in neurogenesis, ensuring the correct functional maturation of newborn neurons. Given the ability of SVZ-derived neuroblasts to target injured areas in the brain, investigating the intracellular mechanisms underlying their motility will not only enhance the understanding of neurogenesis but may also promote the development of neuroregenerative strategies.

This manuscript describes a detailed protocol for the transfection of primary rodent RMS postnatal neuroblasts and the analysis of their motility using a 3D *in vitro* migration assay recapitulating their mode of migration observed *in vivo*. Both rat and mouse neuroblasts can be quickly and efficiently transfected via nucleofection with either plasmid DNA, small hairpin (sh)RNA or short interfering (si)RNA oligos targeting genes of interest. To analyze migration, nucleofected cells are reaggregated in 'hanging drops' and subsequently embedded in a three-dimensional matrix. Nucleofection *per se* does not significantly impair the migration of neuroblasts. Pharmacological treatment of nucleofected and reaggregated neuroblasts can also be performed to study the role of signaling pathways involved in neuroblast migration.

**Figure:**
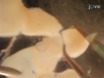


## Introduction

In the postnatal mammalian brain, generation of new neurons (neurogenesis) occurs throughout life and is restricted to two neurogenic niches: the subventricular zone (SVZ) of the lateral ventricles and the subgranular zone of the dentate gyrus of the hippocampus^1^. Several recent studies have shown the important role of adult neurogenesis in facilitating learning and memory tasks^2,3^. Moreover, evidence of proliferation and recruitment of neural progenitors following brain injury^4-7^ raises the possibility of pharmacological activation of neurogenesis in neural repair.

Postnatal neurogenesis is strictly regulated in all its phases, which include neural progenitor proliferation, migration, differentiation, survival, and final synaptic integration of newly born neurons^8^. Neural progenitors (neuroblasts) derived from stem cells in the SVZ migrate over a great distance through the rostral migratory stream (RMS) towards the olfactory bulb (OB) where they mature into functional neurons^9^. Migratory neuroblasts are predominantly unipolar, with an elongated cell body extending a single leading process. These cells move in chains in a collective manner, sliding over one another^10^. Migration is a crucial step for the subsequent maturation of SVZ-derived progenitors into functional neurons^11^ and is controlled by multiple factors and guidance molecules including: polysialylated neural cell adhesion molecule (PSA-NCAM)^12^, Ephrins^13^, integrins^14^, Slits^15^, growth factors^16^ and neurotransmitters^17^, however the molecular mechanisms underlying this process are not fully understood. Investigating the intracellular signaling pathways regulating neuroblast migration will not only provide a better understanding of adult neurogenesis, but will also contribute to the development of the new therapeutic approaches to promote brain repair.

This manuscript describes a detailed protocol to study the role of candidate regulators of neuroblast migration *in vitro* using nucleofection and a 3D migration assay. Nucleofection is a cell transfection technique based on an improved method of electroporation. Cell-type specific electrical current and nucleofection solution allow the transfer of polyanionic macromolecules such as DNA and shRNA vectors and siRNA oligonucleotides directly into the cell nucleus and permit transfection of slowly dividing or mitotically inactive cells like embryonic and mammalian neurons^18^. This method is fast, relatively easy to perform and results in highly reproducible transfection of a broad range of cell types including primary neuroblasts and neurons^19-21^.

Dissociation of RMS tissue allows the isolation of migratory neuroblasts, which can be successfully nucleofected with DNA/shRNA vectors or siRNA oligos targeting genes of interest. Following nucleofection, neuroblasts are reaggregated in hanging drops and subsequently embedded in a three-dimensional Matrigel matrix. These conditions allow neuroblasts to migrate out of the cell aggregates recapitulating the migration mode observed *in vivo*, thus providing an excellent model system to investigate signaling pathways involved in neuroblast migration and to assess the influence of pharmacological treatments on the motility of these cells.

## Protocol

This procedure is in accordance with the UK Home Office Regulations (Animal Scientific Procedures Act, 1986). Scientists should follow the guidelines established and approved by their institutional and national animal regulatory organizations.

### 1. Dissection and Dissociation of Rat RMS Neuroblasts

1. Prepare the solutions required for RMS dissection and dissociation:

**Dissection medium **(100 ml) Hank's Balanced Salt Solution (HBSS) - 98.5 ml 5 M HEPES pH 7.4 - 0.5 ml Penicillin-streptomycin (10,000 units/ml and 10,000 µg/ml) - 1 ml

**Dissociation medium (2 ml)** HBSS - 1.760 ml 10x Trypsin (2.5%) - 200 µl DNAse1 (1 mg/ml) - 40 µl

**Dulbecco Modified Eagle's Medium (DMEM) + 10% Fetal Calf Serum (FCS) (40 ml)** DMEM - 36 ml FCS - 4 ml

**Complete medium** (12 ml)

**Neurobasal medium** - 11.46 ml B27 Supplement - 250 µl L-Glutamine (200 mM) - 125 µl Glucose (45%) - 165 µl

2. Filter-sterilize the DMEM + 10% FCS and the complete medium and preequilibrate them in a 37 °C /5% CO_2_ incubator.

1. Dissection
  1. Sacrifice a P6-P7 rat litter (about 12 pups) by cervical dislocation and decapitate with scissors.
  2. Make an anteroposterior incision in the skin along the mid-sagittal suture from the nose to the cerebellum with a scalpel blade. Peel the skin off and repeat the same incision along the skull.
  3. Gently remove the cranial flaps with forceps and carefully remove the brain with a spatula, taking care to include the olfactory bulbs.
  4. Cut the most caudal third of the brain and discard it.
  5. Chop the brain tissue into 1.4 mm thick coronal slices using a tissue chopper.
  6. Place slices in dishes containing cold dissection medium and carefully separate them using a needle.
  7. The RMS appears as a triangular, translucent area in the centre of OB sections and as a small, circular area in more caudal brain slices. Cut the RMS out of each slice with a microsurgical knife, taking care to avoid including surrounding tissue. In P7 rat pups, usually the ~8 most rostral slices (including OB) contain the RMS.
  8. Collect the RMS fragments with a plastic Pasteur pipette and place them in a small dish containing cold dissection medium on ice.
  9. When the dissection is complete, transfer the RMS fragments into a 15 ml tube with a plastic pipette. Leave fragments to settle at the bottom of the tube.
2. Dissociation
  1. Replace the dissection medium with 2 ml of dissociation medium.
  2. Triturate the RMS fragments by gently pipetting the fragment suspension up and down about 10x using a P1000 pipette.
  3. Leave the tube with the tissue fragments in a 37 °C water bath for 2 min.
  4. Pipette the solution again 10x and ensure that fragments have dissociated (the suspension should become cloudy).
  5. Inactivate the trypsin by adding 5 ml of prewarmed DMEM + 10% FCS.
  6. Centrifuge the cell suspension at 433 x g for 5 min.
  7. In the meantime aliquot the required amount of siRNA/DNA into Eppendorf tubes (usually 3-5 µg DNA/shRNA or 5-9 µg siRNA oligo per nucleofection, however the amount of DNA/siRNA may require optimization).
  8. Remove excess medium and resuspend the cell pellet by gentle pipetting in 5 ml of prewarmed DMEM + 10% FCS.
  9. Perform a cell count. Expect ~1 x 10^6^ cells per rat pup brain. A minimum of 2.5 x 10^6 ^cells are required for each nucleofection, while optimal results are achieved using 3-4 x 10^6 ^cells per nucleofection.
  10. Centrifuge the cell suspension at 433 x g for 5 min. Make sure to **remove as much medium as possible.**

### 2. Nucleofection

1. **Immediately** resuspend the cell pellet in rat (or mouse if using mouse cells) neuron nucleofection solution previously incubated at room temperature. Use 100 µl per nucleofection. *Note*: typically a rat litter of 12 pups is sufficient to perform 4 nucleofections and a mouse litter (12 pups) is sufficient to perform 2 nucleofections.
2. Transfer 100 µl of the cell suspension to each Eppendorf tube containing siRNA/DNA and mix gently 2-3x by pipetting with a P200 pipette.
3. Add the sample (cell-DNA/siRNA suspension) to the bottom of the nucleofectioncuvette, taking care to avoid bubbles.
4. Nucleofect using program G-013 (for rat cells) or O-005 (for mouse cells). One nucleofection takes approximately 5 sec.
5. Quickly add 1 ml of prewarmed DMEM + 10% FCS to the nucleofected sample.
6. Repeat steps 2.4 and 2.5 for all other samples. Note: for optimal results, the entire nucleofection procedure should last no longer than 5 min.
7. Transfer each sample to a 15 ml tube containing 5 ml of prewarmed DMEM + 10% FCS using the plastic pipette provided by the nucleofection kit. Avoid transferring any cellular debris to the tube.
8. Centrifuge samples at 433 x g for 5 min.
9. Carefully remove all excess medium and resuspend the pellet in 25-30 µl of prewarmed DMEM + 10% FCS using a P20 pipette. Do not use more than 30 µl of medium.
10. Pipette the suspension as a drop onto the inner side of a p35 dish lid.
11. Invert the lid over the p35 dish containing 2 ml of complete medium (see also **Figure 1**).
12. Leave in the incubator (37 °C/5% CO_2_) for at least 5 hr and up to 7 hr. Longer incubation time allows better reaggregation of cell clusters.
13. Transfer the hanging drops from the lid into the complete medium in the dish using a P1000 pipette with a cut tip.
14. Incubate at 37 °C/5% CO_2 _for 24 hr for DNA nucleofections and 48 hr for siRNA/ shRNA nucleofections.

### 3. Embedding

1. Prepare complete medium (25 ml) and preequilibrate it at 37 °C/5% CO_2_ for a few hours.
2. Take out the frozen aliquots of basement membrane matrix from -80 °C freezer and thaw on ice in the cold room.
3. For each nucleofection prepare a 6 cm dish containing up to eight 13 mm sterile coverlips.
4. Place the dishes on an ice box covered with cling film. It is important to keep the coverslips cool to prevent matrix solidification during the embedding procedure.
5. To maintain humidity, place a strip of damp tissue inside a 15 cm dish that will be used to hold up to three 6 cm dishes containing the embedded neuroblasts.
6. Add complete medium to the thawed matrix in a 1:3 ratio. For example, mix 40 µl of complete medium with 120 µl of matrix by pipetting. This amount of matrix is sufficient for embedding aggregates on eight 12 mm coverslips.
7. Transfer the reaggregated cell clusters to a 15 ml tube and centrifuge at****433 x g for 5 min.
8. Remove excess medium and resuspend the pellet in 10 µl of complete medium.
9. Place 2 µl of cell aggregate suspension onto each sterile coverslip and add 18 µl of matrix/complete medium mixture. Use the pipette tip to spread the matrix over the entire coverslip.
10. Immediately place the 6 cm dish containing the coverslips in the 15 cm dish and leave in the incubator (37°C /5% CO_2_) for 15-20 min. When the matrix has solidified, gently add 5 ml complete medium to each 6 cm dish taking care to push down any floating coverslip with a pipette tip.
11. Incubate for 24 hr at 37 °C /5% CO_2_ to let neuroblasts migrate out of the cell aggregates.

### 4. 3D Migration Assay

1. Prepare the solutions required for immunostaining.
  1. Prepare the block solution: **Goat block solution (50 ml)** Phosphate Buffered Saline (PBS) - 5 ml Goat serum - 7.5 ml 10% Triton X-100 - 1.5 ml BSA - 50 mg H_2_O - 36 ml
  2. Filter and store at 4 °C.
  3. Prepare the fixing solution: **Fixing solution (100 ml)** Paraformaldehyde (PFA) - 4 g CAUTION: always handle PFA under a hood Sucrose - 20 g (optional) PBS up to 100 ml
  4. On a hot plate and under constant stirring dissolve the PFA in 80 ml PBS maintained at 65 °C.
  5. Once the PFA has dissolved, add 20 g of sucrose.
  6. Adjust pH to 7.4 (usually by adding ~60 µl 1 M NaOH per 100 ml of solution).
  7. Bring up to a total volume of 100 ml with PBS.
2. Immunostaining
  1. Place coverslips in a 24-well plate.
  2. Rinse coverslips with PBS 2x.
  3. Fix the RMS neuroblast aggregates with fixing solution for 45 min at room temperature.
  4. Rinse coverslips with PBS 3x (5 min/wash - on a rocking platform).
  5. Block for 30-60 min with goat block solution.
  6. Dilute primary antibodies in goat block solution and incubate overnight at 4 °C. (If desired, fluorescent phalloidin (1:400) and Hoechst dye (1:10,000) can also be added to the primary antibody solution to visualize filamentous actin and nuclei).
  7. Rinse coverslips with PBS 3x (5 min/wash).
  8. Dilute secondary antibodies in goat block solution and incubate for 2 hr at room temperature.
  9. Rinse coverslips with PBS 3x (5 min/wash)
  10. Mount coverslips with fluorescent mounting medium and leave to dry overnight at room temperature.
3. Migration Analysis
  1. Capture images of fixed RMS neuroblast aggregates with a fluorescent microscope using a 10X objective. Include a scale bar in a sample image.
  2. To set up the scale for quantification, measure the scale bar in the image by selecting the 'Straight line' tool on the ImageJ tool bar.
  3. Choose the 'Analyze' option and click on 'set the scale'.
  4. In the scale window set the 'known distance' and tick the 'global' box to keep the same settings for all measurements.
  5. Use the 'Segmented line' tool on the ImageJ tool bar to measure the distance from the edge of the aggregate to the furthest migrated neuroblast in 6 different sectors around the entire aggregate (**Figure 3B**). Consider only isolated aggregates for analysis.
  6. Calculate an average migration distance from the 6 values obtained for each aggregate.
  7. Measure 10-20 aggregates for each condition in every independent experiment and pool results from a minimum of three independent experiments. Always include a nucleofection control (*e.g*. GFP or a control sh/siRNA).

## Representative Results

Neuroblasts can be successfully isolated from dissected RMS tissue (**Figure 1A**) and embedded in a three-dimensional matrix. Cells isolated from either rat or mouse postnatal RMS are immunopositive for migratory neuroblast markers, such as doublecortin (DCX), βIII tubulin or PSA-NCAM (**Figures 1B-C**). 

Dissociated neuroblasts can be efficiently nucleofected with DNA (*e.g*. a GFP-encoding plasmid, Figure 2) or shRNA plasmids (**Figure 4**) to achieve protein depletion, which can be assessed by western blot analysis (**Figure 4B**) or immunofluorescence (not shown).

Cells nucleofected with GFP-encoding plasmids migrate radially out reaggregated neuroblast clusters (**Figure 3A**). Quantification of relative migrated distance 24 hr post embedding (**Figure 3B**) shows no difference in migration between GFP-positive cells and GFP-negative, nonnucleofected cells (**Figure 3C**), indicating that nucleofection *per se* does not disrupt migration. There is also no significant difference in the extent of migration between nucleofected neuroblasts and neuroblasts directly migrating out of RMS explants (data not shown).


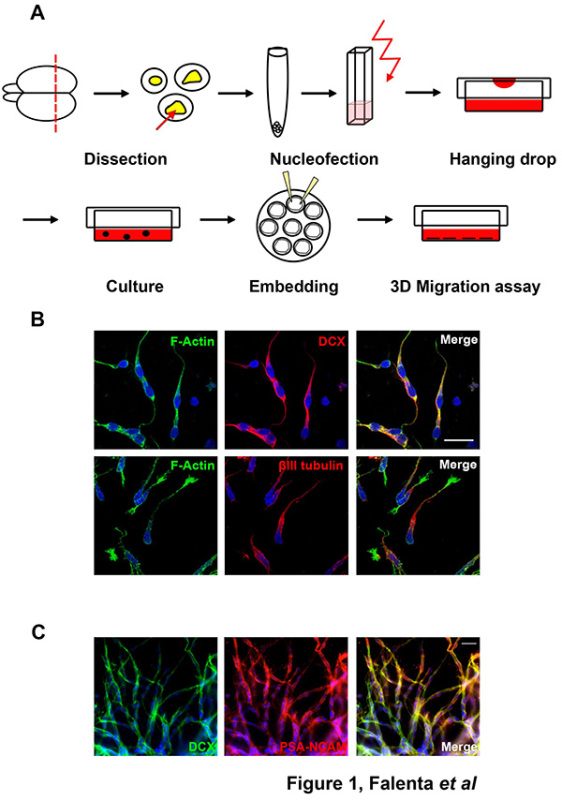
**Figure 1. Dissection of RMS neuroblasts. (A) **Schematic representation of RMS neuroblast dissection. For detailed description please refer to the text**. (B) **Isolated rat RMS cells are immunopositive for the migratory neuroblast makers DCX and βlll tubulin. Bar, 20 µm. **(C)** Cells migrating out of mouse RMS explants express the migratory neuroblast markers DCX and PSA-NCAM. Bar, 20 µm. Click here to view larger image.


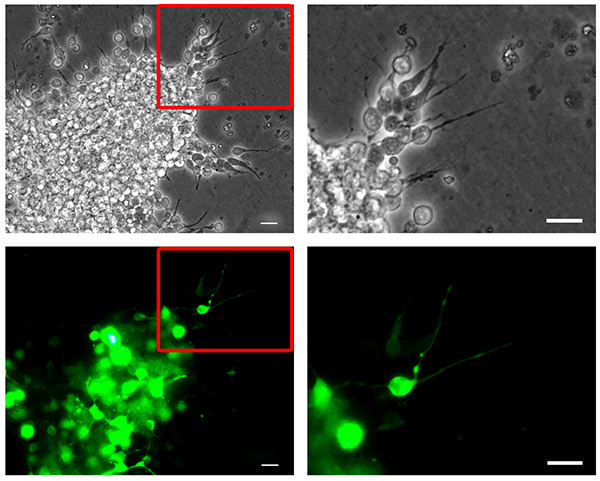
**Figure 2. Mouse neuroblast nucleofection. **Dissociated mouse RMS****neuroblasts were nucleofected with pMAX-GFP, reaggregated, embedded in a three-dimensional matrix and allowed to migrate for 6 hr. Neuroblasts migrating out of a reaggregated cell cluster (top, phase contrast pictures) show high transfection efficiency (bottom, GFP channel pictures). The right column panels show higher magnification pictures corresponding to the insets highlighted in the left column panels. Bars, 20 µm. Click here to view larger image.


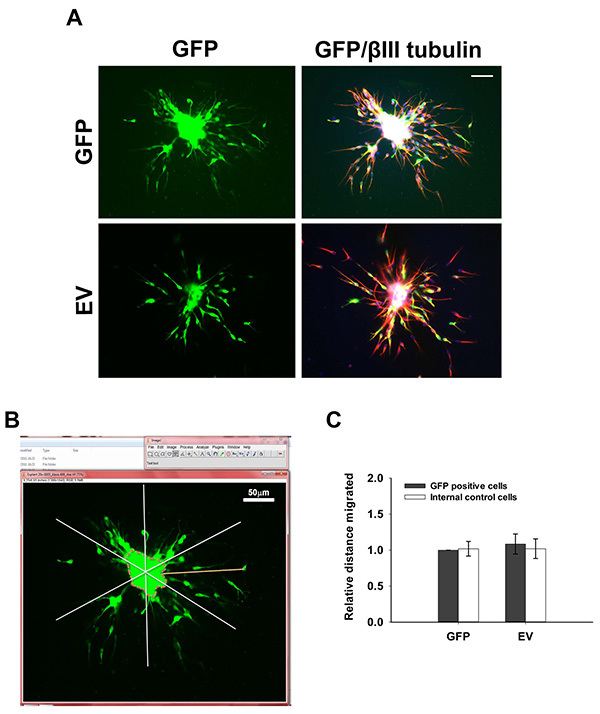
**Figure 3. 3D Migration assay. (A) **Rat neuroblasts were nucleofected with pMAX-GFP (GFP) or pCAG-IRES-EGFP^22^ (EV), reaggregated, embedded in matrix and left to migrate for 24 hr. Cells were then fixed and immunostained for GFP (green) and βIII tubulin (red). Bar, 50 µm. **(B) **Measuring migration distance using ImageJ. The reaggregated cell cluster is divided into 6 equal sectors. The distance between the edge of the cluster (dotted line) and the furthest migrated cell is measured for each sector. **(C)** Quantification of the relative distance migrated by nucleofected cells (GFP-positive) and control, nonnucleofected cells (GFP-negative). Click here to view larger image.


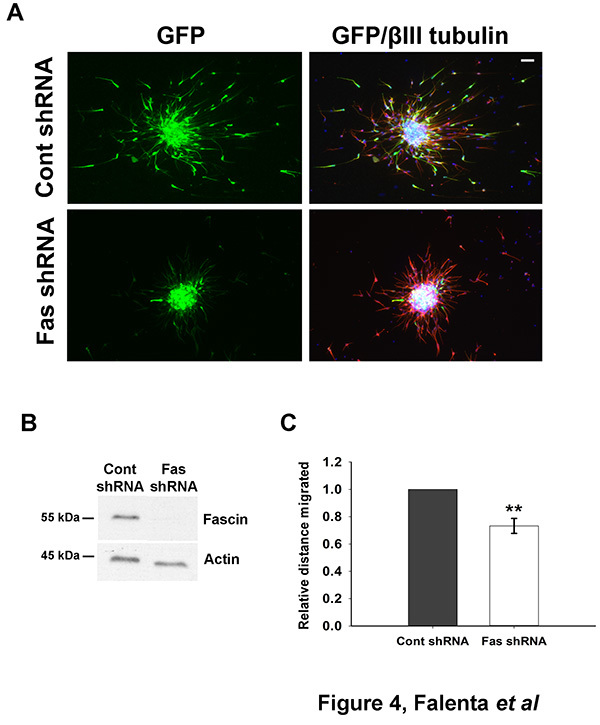
**Figure 4. Monitoring neuroblast migration after shRNA nucleofection. (A) **Rat neuroblasts were nucleofected with a control shRNA vector (pCA-b-EGFPm5 Silencer 3, which also expresses EGFP^23^) or the same vector containing a shRNA targeting fascin, an actin-bundling protein^24^. Cells were reaggregated over 48 hr, embedded in matrix and left to migrate for 24 hr. Aggregates were then fixed and immunostained for GFP (green) and βIII tubulin (red). Bar, 50 µm. **(B) **Effective fascin****depletion can be detected 50 hr after shRNA nucleofection by western blot analysis. Actin is shown here as a loading control. **(C) **Quantitative analysis of relative migration distance showing that fascin depletion significantly impairs neuroblast migration (mean ± SEM; **p<0.01; n=3 independent experiments). Click here to view larger image.

## Discussion

The migration of neuroblasts along the RMS to the final location in the OB is a fundamental step in postnatal neurogenesis. However, the molecular mechanisms controlling this complex process are far from being fully understood.

The experimental procedure described here allows the study of neuroblast migration *in vitro*. We have adapted a previously published protocol for isolating RMS neuroblasts from early postnatal mouse or rat^25^. To achieve optimal results it is important to master the dissection step, since it is crucial to keep the time interval between dissection and nucleofection to a minimum. After nucleofection, neuroblasts can be reaggregated, embedded in a three-dimensional matrix and left to migrate over a 24 hr period. Alternatively, for purposes other than migration (*e.g.* immunofluorescence or western blot analysis), cells can be immediately plated after nucleofection on polyornithine/laminin-coated coverslips, where they survive up to 4-5 days. Mouse and rat neuroblasts migrate in Matrigel to a similar extent, however mouse cells appear to have a stronger tendency to migrate in chains than rat cells.

Depending on the aim of the study, neuroblasts can be nucleofected with different plasmids encoding fluorescent proteins or wild type/mutant proteins of interest. For optimal protein expression plasmids with a CAG promoter (β-actin promoter with CMV enhancer and β-globin poly-A tail)^26^ are highly recommended. Moreover, siRNA oligos or shRNA plasmids can be nucleofected to knockdown targets of interest. Effective protein depletion can be visualized by immunofluorescence or by western blot (usually lysing embedded aggregates from 1 rat pup with 50 µl of standard lysis buffer).

Nucleofection is a relatively simple method to transfect primary neuroblasts, offers an easier and faster alternative to viral vector-mediated transfection, and can achieve high (~70-80%) transfection efficiency. It is critical to work quickly during the nucleofection procedure, since leaving neuroblasts in the nucleofection solution for a prolonged time drastically reduces cell viability.

The average cell yield from RMS dissection is relatively low for P7 mice (~5 x 10^5^ cells/brain) in comparison to P7 rats (~1 x 10^6^ cells/brain) and at least 3 x 10^6^ cells per nucleofection are required to achieve transfection with ~50% efficiency. Moreover, rat neuroblasts appear to resist better to nucleofection compared to mouse neuroblasts. Therefore, early postnatal (P6-P7) rat pups might represent a convenient neuroblast source, also considering that the organization of rat and mouse RMS are remarkably similar^27^ and that the extent of rat and mouse neuroblast migration *in vitro* is also comparable. It is advisable not to keep the reaggregated clusters of nucleofected neuroblasts in suspension for longer than 48 hr to avoid abnormal effects on cell morphology and migration (our unpublished observations).

The 3D assay described here can be used to quantify neuroblast migration at a fixed time point after embedding in matrix (*e.g*. 24 hr). Aggregates of different sizes can be used in the analysis, since there is no significant correlation between the size of aggregates and migration distance (our unpublished observations). To visualize and further investigate the dynamics of neuroblast migration, time-lapse imaging can be used. It is recommended to carry out the migration analysis within a 24 hr interval after embedding, since the speed of neuroblasts appears to drastically decrease at longer time points (our unpublished observations).

There are some limitations to this protocol. First, nucleofection can so far be used for early postnatal rodent neuroblasts, while infection with viral vectors remains the most efficient transfection method for adult neuroblasts^28^. Second, the *in vitro* migration assay does not fully reproduce the complex architecture of the RMS observed *in vivo*. Indeed, although neuroblasts maintain the ability to migrate in a similar way to their *in vivo* counterparts, in the experimental setup described here they lack interactions with other RMS components such as astrocytes and blood vessels, which also contribute to regulate their motility^9,29,30^. This issue may be addressed in the future by optimization of three-dimensional coculture model systems.

In conclusion, combining nucleofection with a 3D migration assay represents a valuable tool to better understand the molecular mechanisms underlying neuroblast migration. This experimental procedure provides an initial, fast and relatively simple method to evaluate the role of candidate regulators of neuroblast migration, which can be further validated by other approaches like *in vivo* postnatal electroporation and time-lapse imaging of brain slice cultures^28,31,32^.

## Disclosures

The authors have nothing to disclose.
